# Investigation of multi-scale spatio-temporal pattern of oldest-old clusters in China on the basis of spatial scan statistics

**DOI:** 10.1371/journal.pone.0219695

**Published:** 2019-07-26

**Authors:** Xin Xu, Yuan Zhao, Siyou Xia, Xinlin Zhang

**Affiliations:** 1 School of Geographic Science, Nanjing Normal University, Nanjing, China; 2 Jiangsu Center for Collaborative Innovation in Geographical Information Resource Development and Application, Nanjing, China; 3 Key Laboratory of Virtual Geographic Environment (Nanjing Normal University), Ministry of Education, Nanjing, China; 4 Ginling College, Nanjing Normal University, Nanjing, China; 5 International Center for Aging and Health Studies (Nanjing Normal University), Nanjing, China; University of the Chinese Academy of Sciences, CHINA

## Abstract

**Background:**

Ageing is becoming a considerable public health burden in China, which produces great societal development challenges. Healthy and active longevity could ease the ageing burden on families and communities. To date, most studies of the oldest-old distribution are focused on a simple scale from spatial perspective, and the multi-scale spatio-temporal clusters trend in the oldest-old population has not yet been determined. Thus, the objective in present study is to use a new method to evaluate the spatio-temporal pattern and detect the risk clusters in the oldest-old population from three scales.

**Methods:**

Individuals aged 65 years or older and individuals aged 80 years or older on three scales in China from 2000 to 2010 were used. The exploratory spatial data analysis was performed using Moran’s I statistic, and the pattern of the oldest-old clusters among humans was examined by using the spatial scan statistical method. Then, spatial stratified heterogeneity was used to explore the factors affecting the spatial heterogeneity of the oldest-old population.

**Results:**

The oldest-old index in the southeast coastal areas is higher than that in the northwest inland areas in China. A three-ladder terrain distribution of the oldest-old index from west to east is obvious. The overall pattern of the oldest-old index evolves from a “concave” shape to an “east-west uplift, and northern collapse” shape. Space-time analysis revealed that high-risk areas were concentrated in five regions: the Yangtze River Delta, the Pearl River Delta, the Southeast Coast, Sichuan and Chongqing, and the Central Plains. The oldest-old cluster at different scales shows a similar pattern, but local differences exist. The risk at the prefecture scale and county scale is greater than at the interprovincial scale; the sublevel can identify clusters that have not been identified at the previous level, especially the bordering areas of prefectures and counties; and more risk units and greater relative risk are found in urban areas than in rural areas.

**Conclusions:**

The results emphasized that spatial scan statistics can be used to estimate the spatial clusters of the oldest-old people. The detection of these clusters might be highly useful in the surveillance of the ageing phenomenon, thus helping local public health authorities measure the population burden at all locations, identifying geographical areas that require more attention, and evaluating the impacts of intervention programs.

## Introduction

China, the most populated country in the world, is experiencing a dramatic increase in its ageing population. Since the reform and opening-up policy, life expectancy (LE) at birth has risen from 69 years in 1980 to 74.8 years in 2010 [1]. During the next 25 years, the percentage of people in China aged 60 years or over is expected to more than double, from 12.4% (168 million people) in 2010 to 28% (402 million) by 2040. Meanwhile, the oldest-old population accounted for 13% of the elderly in 2010, and by 2050 the share will be approximately 30% [2]. This phenomenon is caused by multiple factors: improvement in the socioeconomic status of the population, advances in technology, medical achievement, and China’s “baby boomers”, who were born in the 1950s and 1960s and will be in the “oldest-old” category at that time [3]. A large number of emerging oldest-old people will impose a major challenge for new public health, healthcare, epidemiological, and social care systems [4]. They are the most physically weak group with the most severe disability and morbidity [5]. Their activities of daily living (ADL) [6, 7], health status [8, 9], physical and cognitive function [10, 11], and social support [12, 13] are significantly reduced compared to younger older adults. Reports show that the regular special care and medical and health support demand of the oldest-old is approximately five times that of people 65–79 years old [14], and the average healthcare expense per person among the oldest-old tends to be almost three times higher than the youngest-old [15]. Disability, especially in the oldest-old, represents a limitation on the ability to perform activities of daily living [16]. Prentice and Pizer also pointed out that inadequate access to healthcare may result in the oldest-old being unable to obtain treatment for disease in a timely fashion, which eventually leads to a higher risk of death [17]. Furthermore, chronic diseases, which affect the oldest-old adults disproportionately, contributing to disability, diminishing quality of life (QOL), and increasing health- and long-term-care (LTC) costs, will place an increased burden upon community and healthcare services [18]. Therefore, further clarifying the oldest-old clusters has more important practical significance for regional healthcare resources allocation, nursing intervention policy implementation, public hygiene measures, and healthy ageing.

Several geographers have conducted preliminary exploratory research on the spatial distribution of ageing and longevity. A study by Wang et al. showed that the Shanghai, Zhejiang, and Jiangsu provinces had a high value for the ultra-octogenarian index (the percentage of the population aged at least 80 years) in 2010, which could be considered longevity regions in China. On the other hand, provinces such as Xinjiang and Tibet were the opposite, where the ultra-octogenarian index value was especially low compared to others [19]. Wang et al. found that regions with a higher proportion of the oldest-old were mainly located in the eastern coastal areas, and provinces including Sichuan, Guangxi, Guizhou, Hunan, Hubei and Northeast had a much higher proportion of the oldest-old in 2010 compared with 2000 [1]. Huang et al. revealed that individuals living in southern and eastern coastal regions in China have greater longevity than those in the northern regions [20]. Wang et al. found that there are significant differences between the provinces, urban and rural areas, ethnic autonomous regions and non-ethnic automatic areas, and poor and non-poor areas in the northeast of China [21]. However, these studies only analyzed the spatial differentiation of older adults by traditional spatial methods. The mining of clusters in time and space is not enough, and there is a lack of statistical description for them.

In recent years, with the development of econometrics and analysis technology, the theory and the numbers of models of quantitative analysis clusters have gradually increased; the application of exploratory spatial data analysis (ESDA) is the most common. However, many studies have found that the ESDA method has obvious defects. First, the setting of spatial weight is more subjective and will affect the research results to some extent. Second, the basis of ESDA modeling is completely dependent on spatial cross-section data, without considering the influence of time; however, the space-time interaction is the essential problem for the research agglomeration problem [22]. The spatial scan statistical method overcame the above shortcomings and is well documented in previous literatures. For example, scholars from Switzerland and Bulgaria studied the problems of economic aggregation using the scan statistical method [23, 24]. In disease surveillance, spatial scan statistics are important to detect disease outbreaks [25], such as diabetes [26], tuberculosis [27], leukemia [28], giardiasis [29], and hand, foot and mouth disease [30]. Nevertheless, there are few quantitative and historical studies of the application of the spatial scan statistical method to the heterogeneity of the oldest-old in China. China is a country with a vast territory and abundant resources. The uneven distribution of resources has brought significant differences in regional society and economy, such as medical security, long-term care policy, and the development of healthcare services. Under the background of a new normal ageing society for the oldest-old, clarifying the spatial clusters and its priority at various scales is of great significance to the development of regional health projects and the formulation of public health policies, which could enable governments to formulate relevant policies and health and medical resources allocation planning in time and space.

To fill the gap in the application of spatial scan statistical method in geriatric geography, on the basis of the Fifth and Sixth Census, this study examines the oldest-old clusters from a multi-scale perspective using global Moran’s *I* and the spatial scan statistical method. The research findings provide a social and policy entry-point for the formation of public health polices and healthy ageing strategies.

## Materials and methods

### Data source and selection criteria

#### Demographic data

This study obtained population data from the demographic database for the *Fifth and Sixth National Census* in China, conducted in 2000 and 2010, respectively [31, 32]. The dataset includes the resident population of different age groups at provincial, prefectural, and county levels. The number of residents aged 80 years or older (80+) and 65 years or older (65+) was obtained by calculating and summarizing. In addition, the urban and rural population data are derived from the population of provinces by age and sex in 2000 and 2010 National Census data, with the combination of cities and towns as urban population data.

#### Geographic information spatial data

The basic data comes from the National Geographic Information Resource Directory Service System (http://www.webmap.cn/main.do?method=index). Then based on the 1:1 million geographic information data provided by the former National Surveying and Mapping Geographic Information Bureau in November 2017 (http://bzdt.nasg.gov.cn/), we constructed the vector data of the administrative divisions of the county in 2010 census. Because Chinese administrative adjustments varied frequently from 2000 to 2010, we unified the three scale administrative to make sure both the spatial units and population data comparable between 2000 and 2010.

#### The selection criteria

There was no change in the interprovincial administrative divisions from 2000 to 2010, so this article did not adjust the interprovincial administrative divisions. Considering that the administrative adjustments at prefecture and county levels have been adjusted frequently in previous years, according to the administrative divisions of the 1982–2011 “Administrative Division of the People’s Republic of China”, the vector boundaries and population data for prefectures and counties with arbitrary types of administrative changes from 2000 to 2010 will be merged according to 2010 so that both the spatial units and population data are comparable between 2000 and 2010. Finally, 31 provinces, 354 prefectures and 2328 county administrative units (excluding Hong Kong, Macao and Taiwan) were selected, and the spatial dataset for the population census in 2000 and 2010 was determined (Table 1).

**Table 1:** Information about the geographical units involved and population data.

Provinces (31)	2000population		2010population		Prefectures	Counties
	≥80	≥65	≥80	≥65	*n*	*n*
Beijing	132,928	1,142,864	302,109	1,708,852	1	3
Tianjin	104,077	828,413	204,123	1,102,388	1	4
Hebei	587,760	4,699,148	985,743	5,919,738	11	149
Shanxi	223,128	2,055,048	412,419	2,705,259	11	106
Neimenggu	117,414	1,284,647	241,378	1,868,177	12	87
Liaoning	416,954	3,297,206	800,830	4,509,441	14	58
Jilin	183,742	1,619,759	354,786	2,301,838	8	46
Heilongjiang	200,117	2,015,344	448,938	3,173,314	13	78
Shanghai	298,779	1,880,316	587,804	2,331,313	1	9
Jiangsu	987,438	6,458,388	1,677,416	8,558,646	13	72
Zhejiang	592,594	4,098,584	1,073,027	5,081,675	11	75
Anhui	573,094	4,479,972	1,074,607	6,084,548	16	78
Fujian	335,277	2,279,642	602,500	2,912,130	10	68
Jiangxi	333,563	2,532,328	584,559	3,388,301	11	87
Shandong	1,065,295	7,308,473	1,821,570	9,429,686	17	109
Henan	945,774	6,482,364	1,391,698	7,859,344	18	126
Hubei	448,041	3,818,701	806,669	5,201,894	17	78
Hunan	608,683	4,726,550	1,128,689	6,419,361	14	100
Guangdong	864,645	5,259,948	1,513,703	7,086,150	21	94
Guangxi	526,864	3,202,960	816,004	4,252,921	14	89
Hainan	80,973	509,531	152,966	699,682	18	21
Chongqing	336,170	2,445,382	573,481	3,381,468	1	38
Sichuan	860,724	6,229,433	1,513,094	8,805,507	21	157
Guizhou	272,980	2,102,966	423,582	3,026,181	9	82
Yunnan	317,222	2,580,293	573,757	3,505,474	16	123
Xizang	13,643	124,282	22,906	152,908	7	73
Shaanxi	261,510	2,175,962	421,162	3,183,837	10	96
Gansu	129,535	1,307,498	217,836	2,105,575	14	78
Qinghai	19,581	220,039	37,516	354,684	8	41
Ningxia	27,932	245,501	47,289	402,787	5	19
Xinjiang	124,646	862,480	177,185	1,414,079	11	84
**Total**	**11,991,083**	**88,274,022**	**20,989,346**	**118,927,158**	**354**	**2,328**

### The oldest-old index

The oldest-old index, which is defined as the ratio of individuals aged 80 years or older among the individuals aged 65 years or older, was used to measure the proportion of the oldest-old population [33, 30, 34]. Geographic Information System (ArcGIS 10.0) was used to display the distribution maps of the oldest-old index at county-level.

### Global Moran’s *I*

The overall clustering tendency of the oldest-old index in the study region was assessed by a test of global spatial autocorrelation [35]. This correlation is measured using the Moran’s *I* index, which is a description of the spatial characteristics of ageing in the whole region, and can measure the correlation and difference between regions from a global perspective. High value for *I* implies that the oldest-old index for geographically closer regions are more highly correlated than those from regions that are geographically distant. This method is well known, and the specific formula can be found in the literatures [36, 37].

### Spatial scan statistics

The identification of spatial clusters is a core goal of space science and spatial statistics. Three methods for general, focus, and cluster identification can be used to identify and verify the existence of spatial clusters [38]. Spatial autocorrelation methods such as Global Moran's *I* and Getis Ord Gi* statistics are the most widely used general and focused recognition methods [39]. However, when a spatial autocorrelation method is used, scale selection can be easily influenced by the subjective judgment of the investigator because aggregation is highly scale-sensitive. Moreover, the spatial autocorrelation method does not consider the temporal characteristics of aggregation. The spatial scan statistics developed by Martin Kulldorff can not only detect whether an event agglomerates within a certain area but also accurately locates and scales the characteristics of the cluster from purely spatial and spatio-temporal perspectives; further, this approach determines the priority of the cluster according to the relative risk (RR) [40]. This method has very high specificity in early warning and monitoring of an event and can guide actual work more scientifically. Thus, in the current study, the spatial scan statistical method is used to identify the specific risk clusters of the oldest-old index on three scales.

The principle of spatial scan statistics is as follows: establish a complete study area where a spatial unit is randomly selected as the center of the bottom of the cylinder window; constantly increase the radius of the bottom of the cylinder; change the size of the scan area as the height of the cylinder continues to increase with time and reaches the upper limit set by the scan window. The scanning process is repeated in each study area. Finally, the log-likelihood ratio (LLR) of the test scan statistic is constructed on the basis of the actual number of occurrences and the expected number of occurrences inside and outside the scan window. The window with the highest likelihood ratio is the most likely cluster and is assigned a *p* value through 999 Monte Carlo simulations [41]. The relative risk (RR) and *P*-value also can be calculated for each cluster. The RR value used to measure the risk of the oldest-old index in the cluster is based on how much greater the risk is than outside the window [42].

This study uses the software SaTScan version 9.5 to detect the spatio-temporal clusters of the oldest-old index in China. The output table of SaTScan software was further visualized in the ArcGIS environment [43].

### Stratified heterogeneity analysis

Spatial heterogeneity refers to traits, events, or their uneven distribution between regions. Spatial heterogeneity between strata or areas, each of which is composed of multiple units, is called spatial stratified heterogeneity [44, 45]. We used Q statistics to explore the factors affecting the spatial heterogeneity of the oldest-old index. If a factor and the oldest-old index have significant spatial consistency, this indicates that it has an impact on the formation of the oldest-old spatial differentiation pattern. The value of the *q*-statistics as follows:

$$q=1-\frac{\sum_{h=1}^{L}\sum_{i=1}^{N_{h}}{\left(Y_{hi}-\bar{Y_{h}}\right)}^{2}}{\sum_{i=1}^{N}{\left(Y_{i}-\bar{Y}\right)}^{2}}$$ (1)

where a study area is composed of *N* units and is stratified as *h* = 1, 2…,*L* stratum; stratum *h* is composed of *N_h_* units; *Y_i_* and *Y_hi_* represent the value of unit *i* in the oldest-old index and in stratum *h*, respectively; the stratum mean $\bar{Y_{h}}=\left(1/N_{h}\right)\sum_{i=1}^{N_{h}}Y_{hi}$; and the oldest-old index mean $\bar{Y}=\left(1/N\right)\sum_{i=1}^{N}Y_{i}$. The *q*-statistics are required to be within [0, 1]. The larger the *q*-statistics, the more significance the spatial stratification heterogeneity, with a value of 0 if there is no stratified heterogeneity and 1 if the oldest-old index is completely stratified [46].

## Results

### Spatial pattern of the oldest-old index

**Fig 1:**
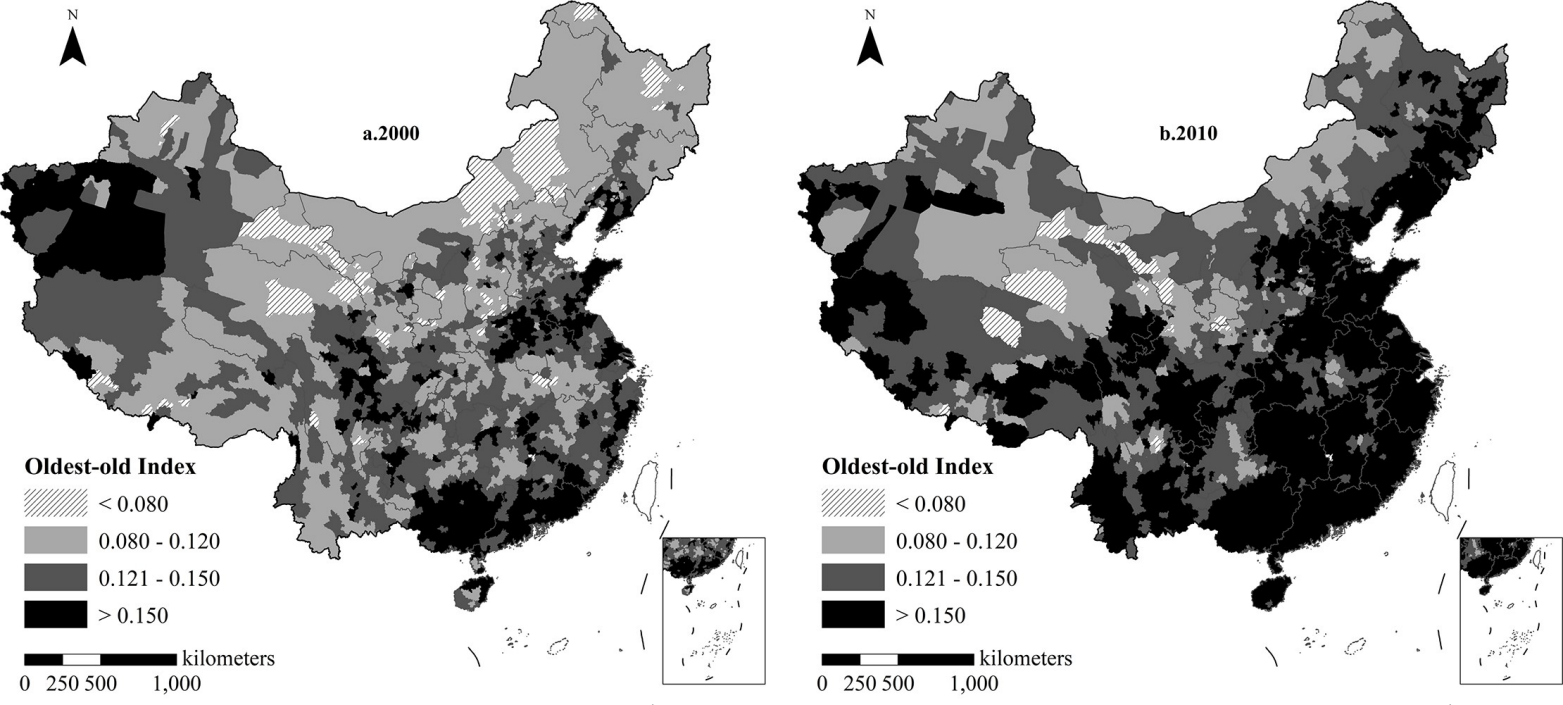
Spatial differentiation pattern of oldest-old index in China, 2000–2010. Figures are drawn by the authors according to the standard map of the National Surveying and Mapping Geographic Information Bureau (Approved drawing number: GS (2016)2893) (http://bzdt.nasg.gov.cn/). All maps on this website are available for free download without copyright.

The oldest-old index in the southeast coastal areas was higher than in northwest inland areas in China from 2000 to 2010 (Fig 1). In the year 2000, the highest oldest-old index appeared in Luopu County (0.293) of Xinjiang province, and the lowest value appeared in Jiayuguan City (0.051), Gansu Province. The Daxinganling, Inner Mongolia plateau, and Jiayuguan areas had the lowest oldest-old index, and 3.91% of the counties’ oldest-old index were lower than 0.080. Areas with lower oldest-old indexes were concentrated in the central and northwest inland areas, which accounted for 35.76%. The vast majority of areas in China had a higher oldest-old index. A total of 894 counties, which accounted for 38.40%, were mostly distributed along the eastern coastline, including the Yangtze River Delta and the Pearl River Delta. A total of 507 counties enjoyed the highest oldest-old index and accounted for 21.78% of the total. These counties were located in Laizhou city of Shandong Peninsula, Tarim Basin of Xinjiang, and some counties south of the Five Ridges. In 2010, the highest value of the oldest-old index was located in Shanghai and the lowest was in Minle County, Gansu Province. The number of regions with a lower oldest-old index decreased sharply, while the number of areas with a higher oldest-old index increased significantly, mainly in the southeast of Hu Line, such as in the Sichuan, Chengdu, Guizhou, Yunnan, Hunan, and Jiangxi provinces. The remaining regions had the highest ageing values.

The three-ladder terrain distribution of the oldest-old index from west to the east was obvious. China is divided into three terrain ladders from west to the east based on different landforms, which are usually referred to as the Western (the first-level ladder), the Central (the second-level ladder) and the Eastern (the third-level ladder). In this study, the oldest-old index of plain hilly and basin areas was generally higher than that of the mountain and the plateau areas. According to the national three-level ladder geomorphology, the spatial stratified heterogeneity was realized by the altitude. The *q*-statistic was calculated to be 0.270 (P = 0.000), which means that spatial stratified heterogeneity existed in the oldest-old people. During 2000, the areas with the highest oldest-old index were concentrated in the Shandong hills, the Yangtze River Delta, the Pearl River Delta, Guangdong and Guangxi hills, and the Tarim Basin areas. During 2010, the ageing areas were basically distributed along the three step junction zone of China's topography. For example, 70.70% of the counties had the highest ageing value and were located in the plain areas, such as the third-level ladder of the plains of the middle and lower reaches of the Yangtze River, the North China Plain, and the Northeast Plain. In addition, 54.41% of areas with lower and higher oldest-old indexes were located in basins, mountains and plateaus regions, such as the second-level ladder of the Yunnan–Guizhou Plateau, the Sichuan Basin, the Loess Plateau, and the Inner Mongolian Plateau. The lowest ageing regions were located in the Qaidam Basin and the Qinghai–Tibet Plateau in the first-level ladder, accounting for 80.65%.

The overall pattern of the oldest-old index evolved from a “concave” shape to an “east-west uplift, and northern collapse” shape. During 2000, the oldest-old index showed a “concave” pattern. Ageing was higher in the eastern and western areas and lower in the northern regions of central China. The pattern of the “east-west uplift and northern collapse” shape become prominent by 2010, and the highest ageing value migrated from the southeast coastal areas and the western marginal zones to the central inland areas. More than 90% of counties in the eastern region had higher oldest-old index by 2010, followed by 60.05% in the central region and 40.58% in the western region, when considering the eastern, central, and western regions as examples.

### Spatial correlation analysis

A strong spatial autocorrelation exists when the value of Moran’s *I* is high. This study calculated the Moran’s *I* value at provincial, prefecture, and county levels (Table 2). Since the spatial weight matrix constructed according to socio-economic relations has the risk of generating multiple collinearity, the distance and k-nearest weight matrix constructed according to the geospatial relationship lacks applicability due to the large difference in the area of each province. Therefore, this paper constructed a simple binary adjacency Queen matrix according to the contiguity criterion, and this standard was also adopted at the prefecture and county level. The global Moran’s *I* values in provincial, prefecture, and county levels from 2000 to 2010 were positive, and the normal statistics *p-value* passed the 5% significance level test, which indicates that spatial autocorrelation existed in the oldest-old index. This finding means that a high-ageing area gathers to a high-ageing area, whereas a low-ageing area gathers to a low-ageing area. Within 10 years, the value of Moran’s *I* increased with fluctuations on three scales. Moran’s *I* increased from 0.4554 to 0.6047 on a provincial scale, 0.5530–0.7048 on a prefecture scale, and 0.6680–0.7397 on a county scale, respectively, which indicates that the cluster degree of the oldest-old index exhibited an increasing trend. Regional differences increased and the uneven development of space was prominent. A comparative study of the same year on three scales found that Moran’s *I* increased from 0.4454 to 0.6680 in 2000 and increased from 0.6047 to 0.7397 in 2010, which means the smaller the scale was, the larger the Moran’s *I* become. Therefore, a small scale is more favorable to reflect the spatial distribution trend of the oldest-old index and the spatial agglomeration of the oldest-old index is obvious on a small scale.

**Table 2:** The estimates of global Moran's *I* of oldest-old index on different scales, 2000–2010.

Scale	Year	Moran’s *I*	*Z*-value	*P*-value
**Provincial level**	2000	0.4554	3.6244	0.001
	2010	0.6047	4.9130	0.001
**Prefectural level**	2000	0.5530	16.0934	0.001
	2010	0.7048	20.9060	0.001
**County level**	2000	0.6680	51.9057	0.001
	2010	0.7397	56.1756	0.001

### Multi-scale spatio-temporal cluster analysis of the oldest-old index

This study used the spatial scan statistical model to analyze the agglomeration of the oldest-old index in 31 provinces, 354 cities, and 2328 county units in the country. Prevalence estimates were used to test for the spatial clusters using the Ordinal model with the purely spatial scan statistics, and to test for the space-time clusters using the Poisson model with the prospective space-time scan statistics. In addition, significant space-time clusters were detected with circular scanning window settings, which included nearly 50% of the population at risk geographically overlapped.

#### Purely spatial analysis

**Fig 2:**
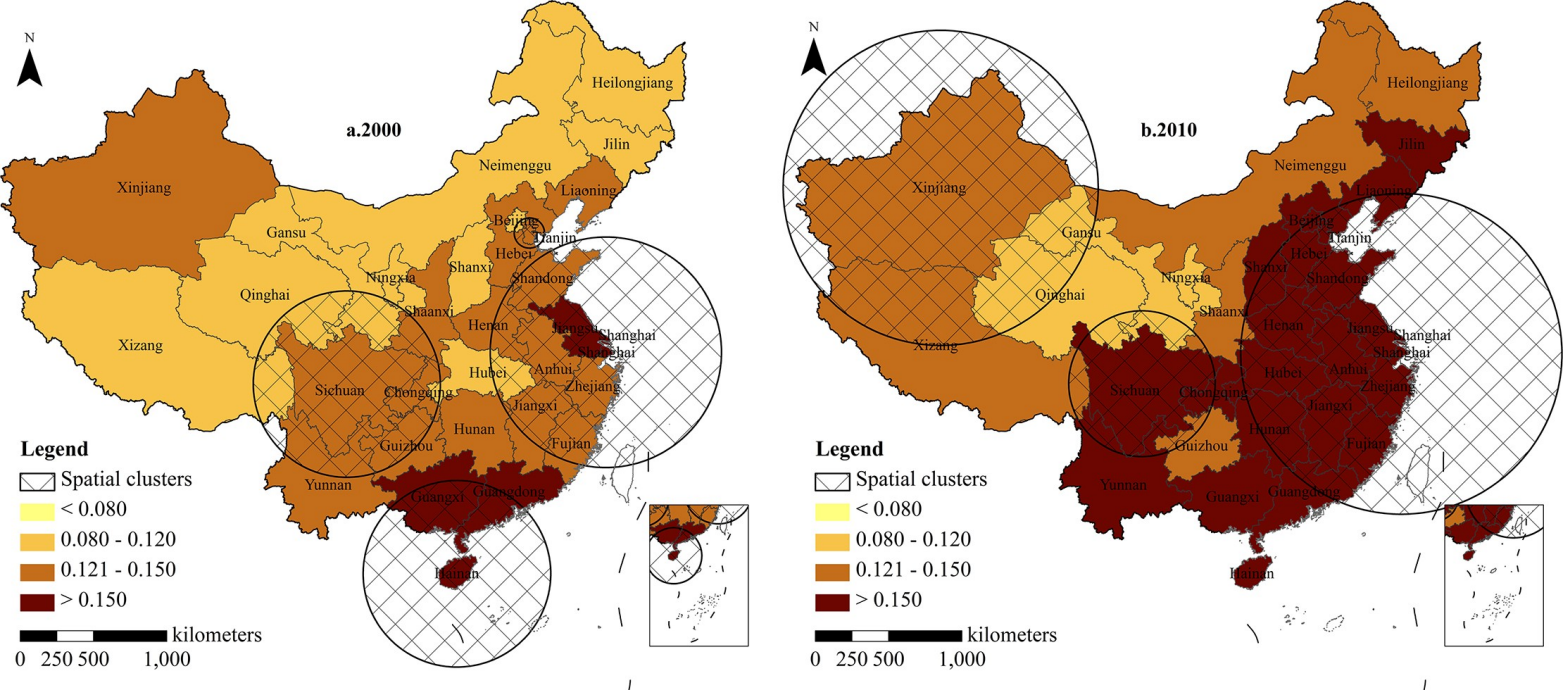
Clusters distribution of oldest-old index in provincial areas, 2000–2010. Figures are drawn by the authors according to the standard map of the National Surveying and Mapping Geographic Information Bureau (Approved drawing number: GS (2016)2893) (http://bzdt.nasg.gov.cn/). All maps on this website are available for free download without copyright.

**Provincial level.** The clusters of the oldest-old index on a provincial level are shown in Fig 2. During 2000, six high oldest-old clusters were detected, with the biggest log likelihood ratio (LLR) being 9,484.19 and the least being 450.00. The biggest relative risk (RR) was 11.26, with the least being 1.44 (P<0.001). The first risk cluster consisted of three provinces: Hainan, Guangxi, and Guangdong (LLR = 9484.19, RR = 11.26). The second risk cluster had the largest coverage and contained eight provinces (Shanghai, Zhejiang, Jiangsu, Anhui, Shandong, Fujian, Jiangxi, and Henan), with the LLR being 4,948.12 and RR being 1.71. Cluster 3 had an LLR of 2,007.28 and an RR of 1.51, which were generally distributed in the Yangtze River Delta and the North China Plain. Cluster 4 included Tianjin and Hebei provinces, with an LLR of 909.39 and an RR of 1.46. Cluster 5 (LLR = 515.46, RR = 1.44) and cluster 6 (LLR = 450.00. RR = 1.44) covered only one province each and were mainly located in Xinjiang and Liaoning Provinces, respectively. By 2010, the number of clusters decreased from 6 in 2000 to 3. The second largest cluster in 2000 became the largest in 2010, with an LLR of 9548.16 and an RR of 1.78. Clusters 2 and cluster 3 encompassed two provinces, respectively. Cluster 2, which was mainly concentrated in the Sichuan Basin in Central China, had an LLR of 881.59 and an RR of 1.31. Cluster 3 was located in Xinjiang and Tibet provinces with the lowest LLR of 183.45 and an RR of 1.07.

The number of risk clusters decreased while the scope expanded from 2000 to 2010, indicating that the oldest-old clusters are concentrated in space. The relative risk (RR) in Shanghai, Zhejiang, Jiangsu, Anhui, Shandong, Fujian, Jiangxi, and Henan provinces increased from 1.71 to 1.78. Hainan, Guangxi, and Guangdong were no longer risk clusters. The RR of Sichuan and Chongqing decreased slightly from 1.51 to 1.31. Guizhou and Yunnan provinces were no longer risk clusters. The RR in Tibet increased and created a new cluster in Xinjiang province.

**Fig 3:**
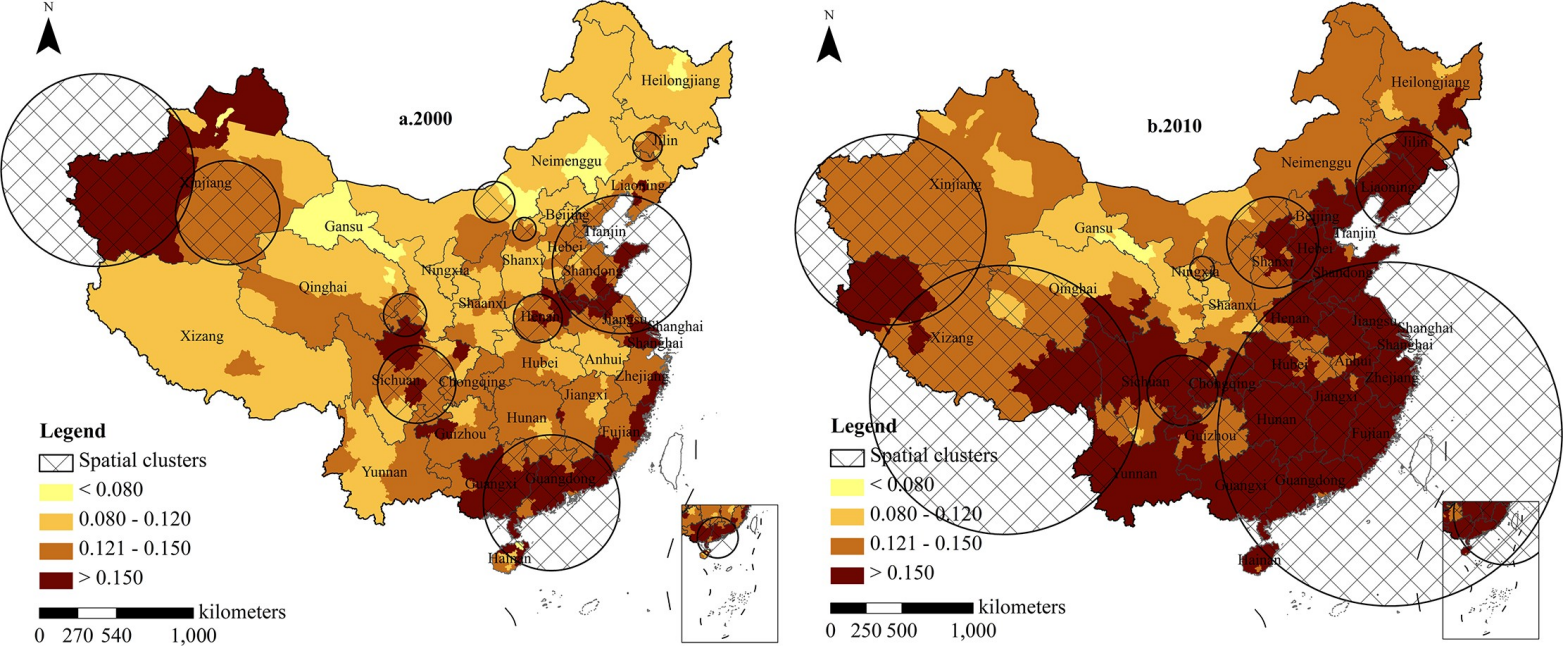
Clusters distribution of oldest-old index in prefectural areas, 2000–2010. Figures are drawn by the authors according to the standard map of the National Surveying and Mapping Geographic Information Bureau (Approved drawing number: GS (2016)2893) (http://bzdt.nasg.gov.cn/). All maps on this website are available for free download without copyright.

**Prefectural level.** The clusters of the oldest-old index on the prefectural level are shown in Fig 3. Regarding the year 2000, 20 high oldest-old clusters were detected with the biggest log likelihood ratio (LLR) being 62,162.11 and the least being 10.74, with the highest relative risk (RR) being 5.05 and the least being 1.01 (P<0.001). Compared to the provincial level, the clusters identified in the prefectural level were widely dispersed and included Guangdong, Guangxi, Hainan, Jiangsu, Anhui, Henan, Shandong, Shanxi, Hebei, Liaoning, Beijing, Tianjin, Inner Mongolia, Jilin, Gansu, Qinghai, Sichuan, Chongqing, Yunnan, and Xinjiang provinces, which indicated that the smaller the scale is, the more dispersed the cluster distribution is and the more accurate the positioning. Although the number of clusters decreased from 20 in 2000 to 13 in 2010, the risk degree of the clusters increased, and regional differences narrowed. In 2010, the cities of Shenyang, Fushun, Changchun, Jilin, Shanghai, Nanjing, Wuxi, Changzhou, Suzhou, Hangzhou, Ningbo, Hefei, Wuhu, Fuzhou, Xiamen, Nanchang, Jingdezhen, Hebi, Wuhan, Huangshi, Changsha, Yueyang, Hechi, Guangan, and Tongren were added to those from 2000. Some newly added cities formed clusters on their own, whereas others formed larger clusters with surrounding cities. However, the RR was generally small and did not increase the country’s risk degree significantly.

From 2000 to 2010, the areas of increased risk were distributed in the northwest half of the Hu Line, such as the Inner Mongolia Plateau, the Northeast Plain, the Qinghai-Tibet Plateau, and Qinghai Province, with an increase of 0.05 to 1.33. The areas of risk reduction were located in the North China Plain, Guangdong and Guangxi hills, the Sichuan Basin, the Tarim Basin, the Qaidam Basin, and the western Inner Mongolia, with a decrease ranging from 0.1 to 5.05. The largest and highest risk clusters in 2000 and 2010 were located in the Guangdong, Guangxi, and Hainan provinces.

**Fig 4:**
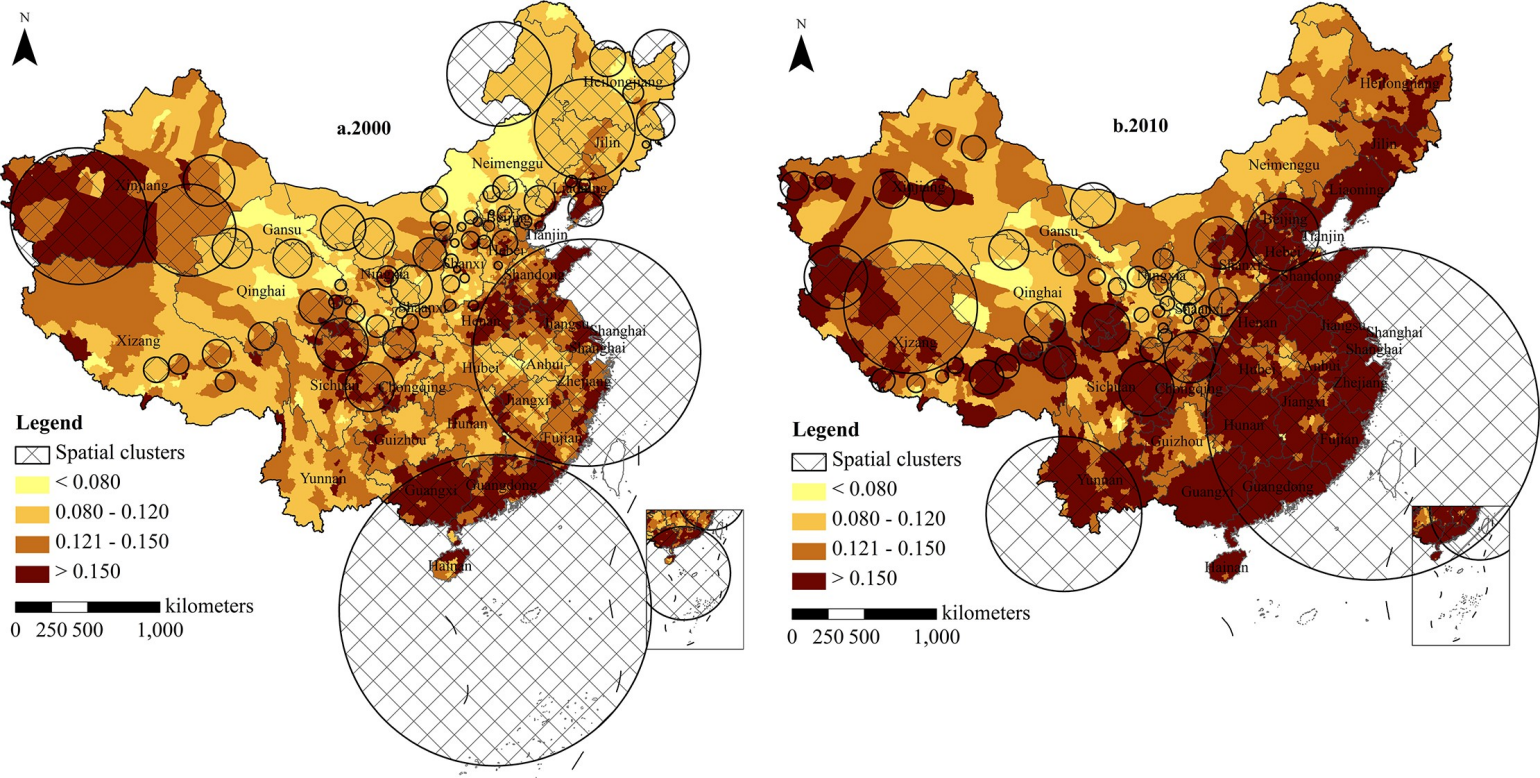
Clusters distribution of oldest-old index in county areas, 2000–2010. Figures are drawn by the authors according to the standard map of the National Surveying and Mapping Geographic Information Bureau (Approved drawing number: GS (2016)2893) (http://bzdt.nasg.gov.cn/). All maps on this website are available for free download without copyright.

**County level.** The clusters of oldest-old index on the county level are shown in Fig 4. A total of 108 clusters covered 1369 county units in 2000. The maximum log likelihood ratio (LLR) was 212,987.81, and the minimum was 16.63. The maximum relative risk (RR) was 3.59 and the least was 1.02. Regarding 2010, the number of clusters decreased to 63, which covered 1491 county units. Compared to 2000, the RR of the oldest-old index in 2010 increased as a whole and the regional differences decreased.

Overall, clusters at the county level were located in the following regions: ①Southern China. Southern China in 2000 had the highest relative risk (RR) in China, which included Dongshan, Nanhai, Sanshui, Qujiang, Pingyuan, and Shaoguan cities and counties in Guangdong province; Guilin, Fengshan, Yizhou, Hezhou, and Beihai cities and counties in Guangxi province; and Wenchang, Qionghai, Chengmai, and Lingao cities and counties in Hainan province. The RR of the above cities and counties were as high as 3.55. The oldest-old indexes in Fengshan, Beihai, Sanshui, and Nanhai were all above 20%. Southern China in 2010 was no longer the highest risk area in China. However, its RR remained higher than 1.60. Compared to 2000, the risk cluster spread eastward in 2010 and was concentrated in Guangdong province, where the oldest-old index was greater than 17%. In contrast, Guangxi and Hainan provinces were no longer risk clusters, and ageing growth had slowed. ②East China. The largest risk cluster in 2000 was located in the eastern coastal region and the middle and lower reaches of the Yangtze River, which included 605 cities and counties in Shandong, Henan, and Jiangsu provinces. Its relative risk (RR) was 1.56, which was lower than in Southern China. The RR in other areas rose to 1.64, and the oldest-old index was greater than 12%. ③Central China and Southwest China, located on the east side of the Hu Line, had the most significant increase in relative risk (RR) from 2000 to 2010. The risk growth was approximately 1.64 in Central China and 1.39 in Southwest China. ④Northwest China. The Tarim Basin in Northwest China became a significant risk reduction area from 2000 to 2010. The RR fell from 3.18 to 0.

#### Space-time analysis

**Scale differences.** The spatio-temporal risk clusters at different scales from 2000 to 2010 are shown in Table 3. Only one cluster existed in China at the provincial scale, with the relative risk (RR) of 9.34 (P<0.001). This cluster included 11 risk units, namely, Shanghai, Jiangsu, Zhejiang, Anhui, Fujian, Jiangxi, Henan, Hubei, Hunan, Guangdong, and Sichuan provinces. Considering the prefectural scale, 135 risk units existed in 12 provinces with an RR of 9.57 (P<0.001). Some cities in Shandong and Guangxi provinces became new risk units, and Sichuan province was no longer a risk unit. Focusing on the county level, 837 risk units were detected in 15 provinces with an RR of 9.56 (P<0.001); many counties in Shandong, Guangxi, Hainan, Chongqing, and Guizhou provinces became new risk units, whereas Sichuan was no linger in the risk unit. The smaller the scale, the more accurate the location. The larger the risk radius, the smaller the scale, which means that more risk units could be detected in a smaller range, and the risk was greater. The oldest-old index risk at prefecture and county scales was greater than that of the interprovincial scale, especially the bordering areas of prefectures and counties.

**Table 3:** Results for spatio-temporal clusters analysis at different scales, 2000–2010.

Scale	Number of regions	Cluster center (latitude/ longitude)	Radius (km)	Observed cases	Expected cases	RR*	*P*-value
**Provincial scale**	11	27.61N, 115.72E	810	11,014,153	1,681,049	9.34	<0.001
**Prefectural scale**	135	26.98N, 119.47E	1055	11,229,042	1,688,730	9.57	<0.001
**County scale**	837	25.53N, 119.76E	1170	11,231,358	1,691,011	9.56	<0.001

*RR: Relative risk

**Urban-rural differences.** The space-time clusters in urban and rural areas are shown in Table 4. One cluster existed in urban and rural areas with a relative risk (RR) of 9.95 and 9.01, respectively. We found that there are more risk units and there is greater relative risk in urban areas than in rural areas. In urban areas, clusters included 11 risk units such as Shanghai, Jiangsu, Zhejiang, Anhui, Fujian, Jiangxi, Henan, Hubei, Hunan, Guangdong, and Chongqing. In rural areas, there were 10 risk units; Guangdong and Chongqing were no longer risk units, and Shandong become a new risk unit.

**Table 4:** Results for spatio-temporal clusters analysis in urban and rural areas, 2000–2010.

Scale	Region	Number of regions	Cluster center (latitude/ longitude)	Radius (km)	Observed/ Expected	RR*	*P*-value
**Provincial scale**	Urban	11	27.61N, 115.72E	810	6.58	9.95	<0.001
	Rural	10	29.10N, 120.08E	836	6.53	9.01	<0.001

*RR: Relative risk

## Discussion

### Spatial distribution of the oldest-old index

This study indicated that the oldest-old index in southeast coastal areas was higher than in northwest inland areas. Consistent with previous literature, the southeast coastal areas, such as the Yangtze River Delta and the Pearl River Delta, are socially economically developed regions. They are also suitable areas for climate, topography and soil and water resources. The appropriate temperature, precipitation, and sunshine provide excellent replacement conditions for the oldest-old to prolong life [47]. Huang et al. also demonstrated that individuals in northwestern China do not live as long as those in eastern and southern China, suggesting that a moderate climate is more conductive to longevity than an extreme climate [48]. We used Q statistics to verify the above conclusion and found that the highest q value was obtained for climate suitability, with a value of 0.282 (p = 0.000); the second highest q value was obtained for topography (q = 0.270, p = 0.000), and the third highest q value was obtained for hydrothermal conditions (q = 0.255, p = 0.000); these are the three main influencing factors on the distribution of the oldest-old population (S1 Table).

The three-ladder terrain distribution of oldest-old index from west to the east was obvious. In western China (the first-level ladder), the main terrain is mountains and plateaus, with an average elevation above 4000 meters. Central China (the second-level ladder) covers both basins and plateaus, having an average elevation between 1000–2000 meters. Eastern China (the third-level ladder) is plain hills, with an average elevation below 500 meters. In China, the plain and basin areas are more conducive to the development of industry and agriculture because of their geomorphological advantages, and these areas are mostly located in the eastern coastal areas, and have good hydrothermal conditions, which is more conducive to the health and longevity of the oldest-old population [49].

From 2000 to 2010, the overall pattern of oldest-old index evolved from a “concave” shape to an “east-west uplift and northern collapse” shape, but this pattern did not cross the Hu Line. This new pattern is more likely to be driven by the economic development of the eastern coastal regions under the diffusion effect. Compared to middle-age elderly, the oldest-old group is special and heterogeneous. They are high-risk people with chronic diseases and disability, and their health status is poor. Under the background of “Ageing before getting rich”, economic security has become the first demand for them [14, 15]. Additionally, the education level also indirectly affects the oldest-old distribution. Generally, the regions with high education level are also relatively developed and their family income and material living standards are high, which helps to improve the health status of the elderly. Furthermore, the oldest-old with a high education level have a strong sense of self-protection and tend to obtain good health information, choose a reasonable diet structure, actively participate in social activities, meet their psychological needs, improve their quality of life, and promote the improvement of regional longevity. The calculated Q values for per capita GDP and the illiteracy rate were 0.028 (p = 0.000) and 0.041 (p = 0.000), respectively, supporting the above conclusion (S1 Table).

### The cluster detection (SaTScan)

Southern China, such as Guangdong, Guangxi, and Hainan, had the highest risk clusters, which is related to a large long-longevity population, dense vegetation, warm and humid climate and a developed economy in these areas [29]. Furthermore, air pollution has become an important factor in life expectancy in the industrial era. Research has shown that air pollution shortened the life expectancy of residents in Northern China by an average of 5.5 years and had increased the incidence of lung cancer, heart disease and stroke [50]. Xinjiang and Tibet have become new ageing clusters, but whether the oldest-old index is true remains to be discussed [51–53]. Some studies had shown that Xinjiang is the province with the largest accumulation of population age structure, because there is a serious age preference phenomenon, namely, people living there prefer to exaggerate their age [54, 55].

We found that the risk of oldest-old clusters at prefecture and county scales are greater than the provincial scale. The sublevel can identify clusters that have not been identified at the previous level, especially in the marginal areas of prefectures and counties. Therefore, the government should regard the county and the city as the focus for the coordination of regional and urban-rural development, considering the impact of social-economic development in the surrounding areas on itself; building a sound social security system and a sustainable development strategy should also be considered.

### Policy recommendations

Different intervention measures should be utilized in different regions. Regarding areas with severe ageing, such as the Yangtze River Delta, the Pearl River Delta, and the Southeast Coast, the base oldest-old population of these areas is large, and the continuous ageing of older adults will apply a great deal of pressure on the local public health system. The oldest-old are most in need of healthcare and assistance; most of them have disabilities in activities of daily living (ADL) and physical and cognitive function impairment [6, 7, 10, 11]. Thus, their need for long-term care is even more urgent. Currently, there are two main options for long-term care: care by family members or care in institutions. However, with the development of industrialization, labor force migration, and the general employment of women in China, the miniaturization of families is becoming more and more obvious. For the future 4-2-1 family structure (four grandparents, two parents, and one child), it is impossible to provide adequate long-term care for the disabled oldest-old. The absence of caregiving beds also poses a great challenge to long-term care [56]. Although China fully implemented a universal “two-child policy” in 2015, this policy will not have an impact on the family structure in the short term [57]. Under such circumstances, government needs to develop a sound long-term care insurance system suited to China’s national conditions in order to cope with the care pressure brought by the oldest-old in different regions and urban-rural areas. Additionally, at the prefecture and county levels, society should build an age-friendly city for the oldest-old, which could effectively support older people within neighborhoods. This approach would require a range of interventions linking different parts of the urban system—from housing and the design of streets to transportation and improved accessibility to shops and services [58].

Central China and Southwest China show the most significant risk increase in the oldest-old index, which means these regions will face a rapid increase in the demand of public health for the oldest-old in the future. The economic development in these areas lags behind that of neighboring Jiangsu and Zhejiang provinces and the climate conditions are not as good as those of Guangdong and Guangxi provinces [47]. The increasingly oldest-old risk clusters in the local area, in this context, should attract more attention from the government to vigorously develop the economy and improve the public health demand and long-term care services, thus prolonging the life expectancy of the oldest-old. Specifically, changes and development in healthcare should be consistent with the oldest-old change and development, including quantitative and structural consistency. The consistency of quantity refers to various health resources, such as health institutions, health workers, hospital care beds, and medical equipment, which must maintain a moderate growth rate to meet the needs of the oldest-old growth. Structural consistency means the development of various types of health resources. The internal structure and layout should be consistent with the requirements of the oldest-old. For instance, with the increase in the oldest-old, the mortality rate, especially various chronic disease mortality (cardiovascular and cerebrovascular disease), is further increased. Therefore, the demand for health services, such as chronic disease prevention and rehabilitation medicine, will increase. Under such circumstances, it is necessary to vigorously develop medical and health institutions, such as chronic disease prevention and treatment centers and rehabilitation hospitals, and to expand the coverage of community medical care to meet the needs of health services brought by the oldest-old.

Urban areas have more risk units and relative risk than rural areas, which is related to the differences in socioeconomic status and access to healthcare system brought by the urban-rural dual-system. For urban residents, the existing healthcare insurance is employment-based, including the Urban Employer-sponsored Medical Scheme (UEMS) and the Urban Resident Medical Scheme (URMS); for rural people, the current health insurance is the New Cooperative Medical Scheme (NCMS) [59, 60]. This disparity affect the health and longevity of older adults to some extent. Currently, severe inequality exists in health services between urban and rural areas, with 80% of health resources (hospitals and healthcare workers) allocated to cities [61]. Older adults from rural areas in China are less likely to use hospitalization services and rely more on family health services than urban older adults [60]. This preference leads to a difference in urban and rural mortality, further affecting the distribution of longevity population regionally. Previous studies have reached similar conclusions [17, 62]. Thus, the government should make great efforts to reform China’s healthcare insurance system, achieving universal health insurance so that everyone can enjoy fair medical insurance benefits. Additionally, education is representative of social and economic status, and highly educated patients are likely to be employed and, therefore, insured. However, most rural residents in China are poorly educated and have no insurance [63]. Steps to improve their education level are essential to improve regional social security.

### Contributions and limitations

The contributions of this paper lie in the following aspects: 1) First, the authors proposed an approach for detecting oldest-old clusters in both temporal and spatial dimensions, which will provide additional information necessary to improve the health of the oldest-old. As we all know, public health authorities often need to respond to request to investigate potential clusters of different diseases. However, due to the complexity and cost of cluster research, they are usually unable to thoroughly investigate all potential clusters [29]. Thus, using the cluster survey statistical method, health managers will be able to determine the clusters with statistical significance and the priority clusters that need to be investigated. 2) Second, this study first used a spatial scan statistical method to study the oldest-old clusters in different geographic units that had not been studied in the existing literature. This research is of great practical significance. On the one hand, exploring the oldest-old pattern at various scales is beneficial for governments at all levels (countries, regions, locals) to formulate strategies to address ageing challenges and find the focus of population structure optimization; on the other hand, we want to determine which scale is the best to understand the oldest-old proportion in China. 3) Finally, this method makes an important contribution to the theoretical knowledge of geriatric geography, which can be used as a reference for future research.

However, there are also some limitations to this research. The risk of the oldest-old is divided into macro and micro risks. The authors used the ratio of individuals aged 80 years or older among the individuals aged 65 years or older to measure the oldest-old risk from a macro perspective, which reflects the challenges brought by the increase in the oldest-old to public health, long-term care, and the allocation of medical and health resources. However, this indicator does not reflect the health risks of the oldest-old as individuals from their living conditions, chronic diseases, health care and subject choices. Thus, in future research, a relevant study should be added to make the conclusions more comprehensive.

## Conclusions

This study emphasized the application of spatial scan statistics as a new method to explore geographical variation and extended descriptive statistical analysis of the oldest-old at a specific period. Under the context of global public health and socioeconomic sustainable development, this method could provide public health managers with additional tools for ageing surveillance. More detailed social surveys are needed in identified clusters to determine the most important determinants of the distribution of the oldest-old and to address the social burden that may arise.

## Supporting information

S1 File Province population in 2000 and 2010. S1 File contains details at Provincial Level of China in 2000 and 2010 in terms of Province name in which it is located; population data such as the individuals aged 80 years or older (80+) and the individuals aged 65 years or older (65+); geographic information such as longitude and latitude; and some TXT files for spatial scanning.
(RAR)

S2 File Prefecture population in 2000 and 2010. S2 File contains details at Prefecture Level of China in 2000 and 2010 in terms of Prefecture name in which it is located; population data such as the individuals aged 80 years or older (80+) and the individuals aged 65 years or older (65+); geographic information such as longitude and latitude; and some TXT files for spatial scanning.
(RAR)

S3 File County population in 2000 and 2010. S3 File contains details at County Level of China in 2000 and 2010 in terms of County name in which it is located; population data such as the individuals aged 80 years or older (80+) and the individuals aged 65 years or older (65+); geographic information such as longitude and latitude; and some TXT files for spatial scanning.
(RAR)

S4 File Provincial urban and rural population data in 2000 and 2010. S4 File contain urban and rural details at Provincial Level of China in 2000 and 2010 in terms of Province name in which it is located; population data such as the individuals aged 80 years or older (80+) and the individuals aged 65 years or older (65+); geographic information such as longitude and latitude; and some TXT files for spatial scanning.
(RAR)

S1 Table Q statistics of driving factors on county-level in 2010. (XLSX)
